# The degree of association between overweight and obesity with the use of electronic media among Bangladeshi adolescents

**DOI:** 10.1371/journal.pone.0280544

**Published:** 2023-01-20

**Authors:** Suvasish Das Shuvo, Biplob Kumar Biswas

**Affiliations:** Department of Nutrition and Food Technology, Jashore University of Science and Technology, Jashore, Bangladesh; Universiti Malaya, MALAYSIA

## Abstract

**Background:**

Electronic media usage is recently considered a modifiable risk factor for overweight and obesity among adolescents. The purpose of this present study was to evaluate the association of electronic media (EM) usage with overweight and obesity among school-going adolescents.

**Methods:**

This cross-sectional study was conducted from October to December 2019 among school-going adolescents (14–16 years old) residing in the Jashore Sadar Upazila, Jashore district of Bangladesh. A standardized questionnaire was used to collect information regarding the socio-economic status, time spent watching television, video games playing, computer, and smart mobile phone use through face-to-face interviews. Age- and sex-specific body mass index (BMI) cut-off values for overweight and obesity were determined for Asian adolescents by the International Obesity Task Force (IOTF). Multinomial logistic regression analysis was carried out to determine the association between electronic media use with overweight and obesity.

**Findings:**

The findings suggest that the overall prevalence of overweight and obesity was 13.5% and 25.2%, respectively. Among the total adolescent students, about 49.1% highly (above 3 hours per day) spent their time on EM use whereas 30.6% moderately (≥121 to 180 min/day) use EM. The regression analysis showed that spending high time using total screen-based electronic devices, television viewing, video game playing, computer use, and smartphone use were significantly associated with overweight (RRR: 7.36, 95% CI: 3.64–11.54; RRR: 4.58, 95% CI: 1.46–7.95; RRR: 4.45, 95% CI: 2.75–6.12; RRR: 3.18, 95% CI: 1.87–4.70; RRR: 2.15, 95% CI: 1.23–3.51) and obesity (RRR: 8.72, 95% CI: 4.64–12.54; RRR: 2.89, 95% CI: 1.31–5.21; RRR: 3.88, 95% CI: 1.74–5.13; RRR: 3.08, 95% CI: 1.32–4.86; RRR: 1.19, 95% CI: 0.93–1.48) in adolescents, respectively.

**Conclusion:**

The results support the total time spent using electronic media was associated with an increased risk of being overweight and obesity. Finally, this study strongly suggests the proper use of electronic media may be necessary to reduce the risk of being overweight and obesity in early adolescents.

## 1. Introduction

Adolescence is a significant period (from ages 10 to 19) of human life with major formative encounters introducing the much-expected move from childhood to adulthood [1]. The well-being of children and teenagers is of most extreme significance to each country, as they are respected as one of the basic determinants of future economic and societal development [1]. Globally, the prevalence rate of overweight and obesity in adolescents is increasing rapidly and is a major alarming public health threat in both developed and developing countries [2]. Obesity and overweight are characterized as abnormal or excessive fat accumulation, which is primarily caused by an imbalance between energy intake and expenditure and may lead to impaired health [3]. According to World Health Organization (WHO), above 158 million children and adolescents were considered obese in 2020 [3] whereas nearly 81% were reported from developing countries [4, 5]. It is also projected that the prevalence of overweight and obesity will touch 254 million by 2030 around the world [3]. The increasing rate of overweight (9.5%) and obesity (3.5%) is also a concerning issue in Bangladesh [6, 7]. Shreds of evidence from some studies also explain that the double burden of malnutrition (DBM) is an emerging public health issue in Bangladesh nowadays whereas undernutrition is a predominant burden with the escalating burden of overweight and obesity [8]. In recent years, sedentary behavior is becoming a key factor in obesity, and it should be recognized as a separate behavior from physical activity [9].

Sedentary behavior is defined as sitting or reclining during waking hours when energy expenditure is less than 1.5 metabolic equivalents [10]. Although there are various forms of sedentary behavior, the most prominent is screen-based electronic devices (e.g., watching television or using a computer and smart mobile phone), which are frequently used as a proxy indicator of sedentariness among adolescents [11]. In recent years, sedentary behaviors play a significant role in increasing the risk of overweight and obesity among adolescents [9, 11, 12]. Several epidemiological studies and meta-analyses have suggested that all sedentary behaviors including television viewing, videogame playing, computer, and smartphone use are related to an increased risk for overweight and obesity [11, 13, 14]. Different studies proposed that the possible mechanism for overweight and obesity because of using electronic media (EM) are displacement of physical activity, reduction in resting energy expenditure compared to other activities, increased sleep deprivation, exposure to advertising, and consequent use of foods commonly advertised on television, and increased calorie intake [14–16]. Additionally, It was hypothesized that the use of EM might displace the amount of strenuous physical activity time with the increased sedentary activities [14, 16–18]. Besides advertisements telecast during programs on television also increase the risk of overweight and obesity [17, 18]. Recent empirical studies reveal that computer use and video game playing was associated with the risk of obesity among adolescents [14, 17, 19]. But the evidence of a relationship between smartphone use and the risk of overweight and obesity is still limited [14, 16].

Furthermore, the regular use of EM in adolescents’ recreational and academic daily life is increasing gradually which may result in an escalation of obesity [9, 13, 20]. Problematic social media use is impacting an increasing number of adolescents’ daily activities, academic achievement, family ties, and emotional regulation [21]. Different studies also explored that heavy use of EM decreases physical activity time in youth adolescents which may be a potential risk factor for higher Body Mass Index (BMI) in youth adolescents [11, 15, 22]. Adolescent sedentary behavior can persist into adulthood and contribute to the development of chronic disease later in life and is well-recognized to associate with co-morbidities [23]. Additionally, numerous studies have identified overweight or obesity among adults as a strong predictor of the development of premature death and chronic Non-communicable Diseases (NCDs) including type-2 diabetes, hypertension, cardiovascular diseases, and cancer [24–26].

However, several studies have identified the effects of excessive screen-based electronic device (EM) use on the risk of overweight/obesity in adolescents which do exist have mainly been undertaken in both developed and developing countries [13–16, 22]. Evidence on the relationship between EM use and obesity among young adolescents in Bangladesh perspective is hardly available. Likewise, very few studies have investigated gender differences and screen-based behaviors of adolescents [13, 27]. In Bangladesh, rapid socio-economic growth and increasing urbanization can be associated with an increase in sedentary behavior. Recently, adolescents are being engaged at a great rate in technological interactions (e.g., social networking via Facebook, and Twitter) and screen-based activities (e.g., television, and smartphone), which are sedentary in nature [27]. Since the concurrent usage of various electronic screen devices has become an emerging trend in Bangladesh, a comprehensive investigation of the usage of several EM may help us to understand the overall situation. Therefore, the purpose of this study was to evaluate the association between electronic media use and BMI among Bangladeshi adolescents.

## 2. Methods

### 2.1 Study area

This study was conducted at Jashore *Sadar* Upazila, Jashore district of Bangladesh, which is an administrative town of the Jashore district, also the center of the district. Jashore *Sadar* Upazila area is 435.41 square kilometers, located between 23°04’ and 23°20’ north latitudes and between 89°06’ and 84°06’ east longitudes [28]. The total number of adolescents residing in the urban areas of the *Sadar* district was 150462 [28]. Overnutrition is one of the main health problems among adolescents in the southwestern regional state. It is predominantly seen among the urban population, nearly 19% and 11% of adolescents are overweight and obese [6]. Recently, adolescent obesity is a major recent concerning study issue in Bangladesh. Thus, we selected the study area.

### 2.2 Study design and sample selection

**Fig 1:**
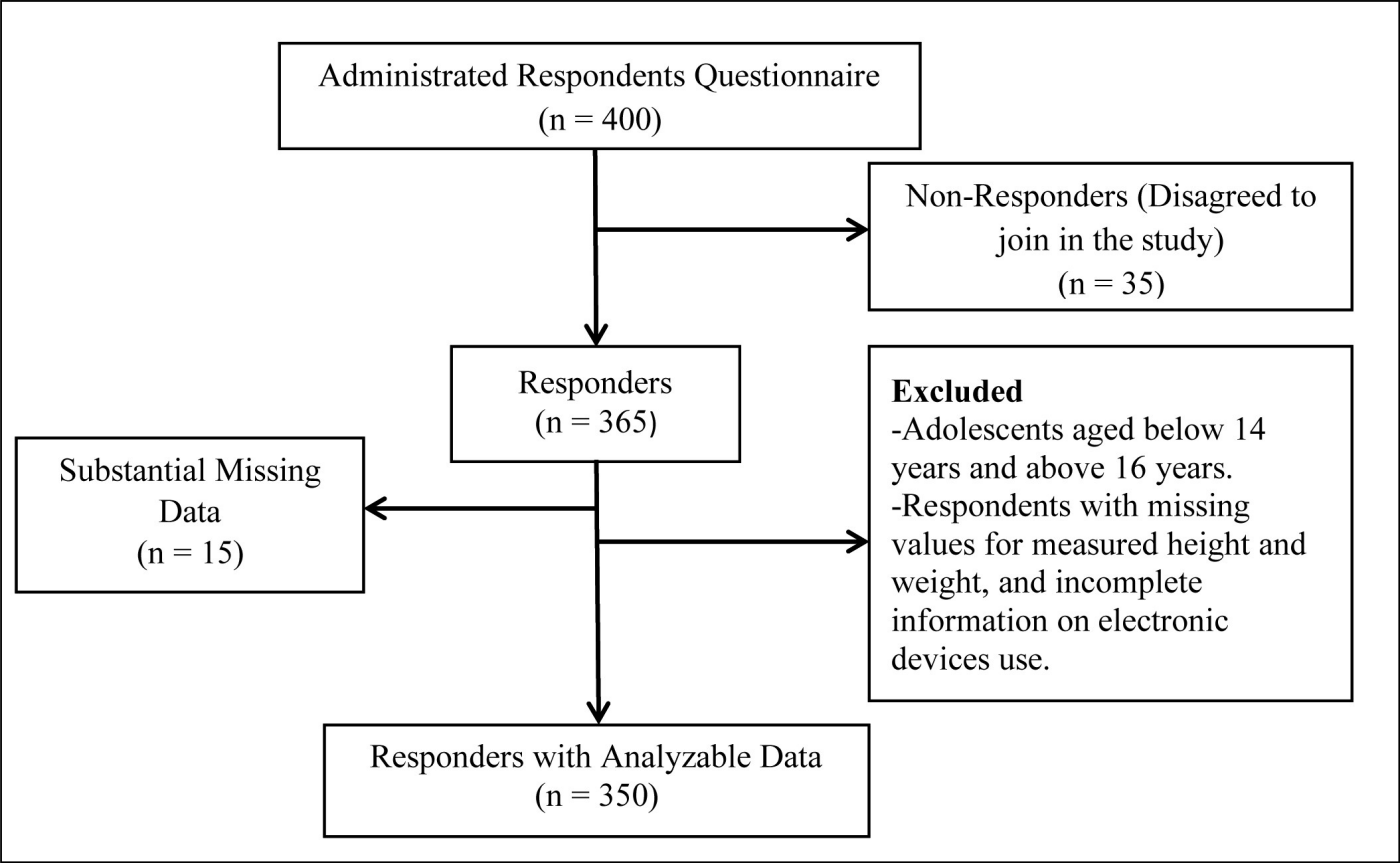
Flow chart of respondent’s recruitment.

A cross-sectional study was conducted among Bangladeshi secondary school adolescents residing in the Jashore Sadar Upazila, Jashore district of Bangladesh. The respondents of class eight and nine grade students aged between 14 to 16 years old were included in this study. The estimated sample size of 246 was determined by using formula Z_1-α/2_^2^p(1-p)/d^2^ with the previously published studies prevalence of overweight and obesity [6] (considering 20% prevalence), 5% margin of error to be tolerated at the 95% level of confidence, and 95% response rate. Hence, N = Z^2^p(1-p)/d^2^ = (1.96)^2^ × (0.20) × (1–0.20)/(0.05)^2^ ≅ 246. Although 400 respondents were approached, only 350 eligible respondents agreed to participate. Adolescents aged below 14 years and above 16 years were excluded from this study. As well, respondents with missing values for measured height, weight, and adolescents with incomplete information on electronic media use at the time of the survey were also excluded (Fig 1). This survey includes secondary educational institutions from three places (*Jashore Jila School*, *Govt*. *Momin’s Girls School Jashore*, and *Madhusudan Taraprosonno Girls School*) in urban areas of the Jashore Sadar Upazila, Jashore District. A two-phase cluster sample was designed to collect the respondents. Firstly, the probability of secondary schools being selected was proportional to the number of students in the specified grades and secondly, all the respondents were selected by a simple random sampling method from the selected classes.

### 2.3 Data collection and study variables

A face-to-face interview was conducted by the four trained interviewers who had academic and research experiences in the related field were enrolled to collect the data. Data were collected between October and December 2019, during regular school hours in the presence of the classroom teacher and by trained interviewers. A validated standardized survey questionnaire was firstly designed in the English language then translated to the native language (Bengali) and again re-translated back to English to check for its uniformity. A pretested questionnaire was asked of the school adolescents to collect the data. The purpose of the study was clearly explained to adolescents and those who agreed to join the study were asked to sign an agreement form.

#### 2.3.1 Socioeconomic variables

Socioeconomic data of the respondents were obtained through closed-ended questions including their gender, grade of school, unhealthy eating habits during electronic media use, physical exercise, parent’s educational level, occupation, and monthly family income. Monthly family income was categorized into four classes: ≤10,000 Bangladeshi Taka (BDT), 10,001–20,000 BDT, 20,001–30,000 BDT, and >30,000 BDT.

#### 2.3.2 Outcome variable

This study mainly focused on the outcome of overweight and obesity based on the screen time used by respondents. The survey collected an individual’s measurement of weight and height to calculate the Body Mass Index (BMI) [BMI = weight (in kilograms) / height (in meters squared)]. According to the International Obesity Task Force e (IOTF) using age- and sex-specific BMI cut-off points for overweight and obesity for adolescents are defined as 25 kg/m^2^ and 30 kg/m^2^, and 23 kg/m^2^ and 25 kg/m^2^ for Asian children and adolescents, respectively [29, 30]. The function *zbmicat* in Stata was used to categorize adolescents as obese, overweight, or normal weight. In this analysis, since underweight was not the main concern, it was merged with the normal group to derive a new category (BMI ≤ 22.9), considered normal that was used as a reference in logistic regression analysis [29]. To evaluate the risk ratio, these outcome variables were coded: 0 for normal weight, 1 for overweight, and 2 for obesity.

#### 2.3.3 Exposure variables

The main variables of interest in the present study were daily television, video game, computer, smartphone, and total electronic media use. Electronic media (EM) use time data collected from each adolescent by using the Multimedia Activity Recall for Children and Adolescents (MARCA) 24-hour activity recall diary [31, 32]. Respondents were requested to complete MARCA dairies’ school days and or another day (weekend, holiday, or day off from school) with the help of an interviewer. Adolescents who did not give the impression to make a good effort at recall were excluded from the analysis.

The MARCA’s analytical software module was used to estimate minutes (per recall) regarding television watching, computer use (for typing, Internet), video games playing (via computer, other e-games), and smartphone use (talking/messaging/watching video). This study categorized total and individual electronic media use into four quartiles including none, low, medium, and high users according to specific electronic media use time for consistency [23, 24]. The use of the computer for video game playing does not include in the category of computer use which was considered within the video games category. The average daily time spent in EM use was calculated in both school and non-school day recall which was again calculated as the average of the average school and non-school day recall [32, 33].

### 2.4 Estimation strategy

Descriptive statistics (frequency and percentages) were performed for the explanatory variables by outcome variables, and the prevalence of BMI within different categories of a variable was assessed using the Chi-square test. Multinomial logistic regression models were used to explore the association of electronic media use with overweight and obesity. Average daily media use, computer, video game, and smartphone use were selected as a covariate in the logistic regression analysis for the continuous outcomes (overweight and obesity). The model was adjusted with gender, grade of school, eating habits, and physical exercise variables. Results were presented with a relative risk ratio (RRR) and 95% confidence interval. Categorical independent measures were entered into the model using categorical variables to compare the effect between the first category (reference) and each other category. The potential collinearity of the predictor variables and the outcome variable were examined using the Variance Inflation Factors (VIFs) before fitting the models. The VIFs for all covariates that were included in the multinomial logistic regression analysis were less than 2.0 [34]. All statistical analyses were conducted using Stata version 14.0 and the *p*-value was set at <0.05 level for statistical significance.

### 2.5 Ethical considerations

Ethical approval and prior permission were obtained from the institutional Ethical Review Committee of the Faculty of Biological Science and Technology, Jashore University of Science and Technology, Bangladesh (Ref: ERC/FBST/JUST/2021-60). All the study respondents formally consented before their participation and signed consent was obtained for each participant. Additionally, consent from also obtained from all parents of the minors. Keeping confidentiality in data collection, the data collector, supervisor, and investigator used code numbers instead of the student’s name.

## 3. Results

Table 1 shows the socio-demographic status of the study participants. Among 350 adolescents, 60.8% were boys and 39.2% were girls. Approximately, 57% were studied in class nine and 43% were in class eight. Overall 51.7% of adolescents had unhealthy eating habits during electronic media use and 62.4% did not regularly perform physical exercise. Regarding parents’ education level, 44% and 62% of respondent’s mothers and fathers received graduate degrees, while 13.2% and 10.9% of the adolescents’ mothers and fathers had completed primary education. In terms of parents’ occupation, about 78% of adolescents’ mother was housewives and 40% of adolescents’ father was businessmen while nearly 4% was day laborers. A total of 39% and 37% of the adolescents’ monthly family income were above 30,000 BDT, and between 20,001–30,000 BDT.

**Table 1:** Background characteristics of study participants.

Variables	Categories	Frequency (%)
Gender	Boy	213 (60.8)
	Girl	137 (39.2)
Grade of school	Eight	150 (42.9)
	Nine	200 (57.1)
Unhealthy eating habits during electronic media use	Yes	181 (51.7)
	No	169 (48.3)
Physical exercise (≥30 min/day)	Yes	132 (37.6)
	No	218 (62.4)
Mother’s educational level	Primary	46 (13.2)
	Secondary	74 (21.1)
	Higher Secondary	76 (21.7)
	Graduation degree	154 (44.0)
Father’s educational level	Primary	38 (10.9)
	Secondary	45 (12.8)
	Higher Secondary	50 (14.3)
	Graduation degree	217 (62.0)
Mother’s occupation	Housewife	271 (77.4)
	Govt. Employee	38 (10.9)
	Private Employee	31(8.8)
	Business	10 (2.9)
Father’s occupation	Govt. Employee	89 (25.6)
	Private Employee	86 (24.7)
	Business	160 (40.0)
	Day labor	13 (3.7)
Monthly family income (BDT)	≤10,000	24 (6.9)
	10,001–20,000	61 (17.4)
	20,001–30,000	130 (37.1)
	>30,000	135 (38.6)

**Fig 2:**
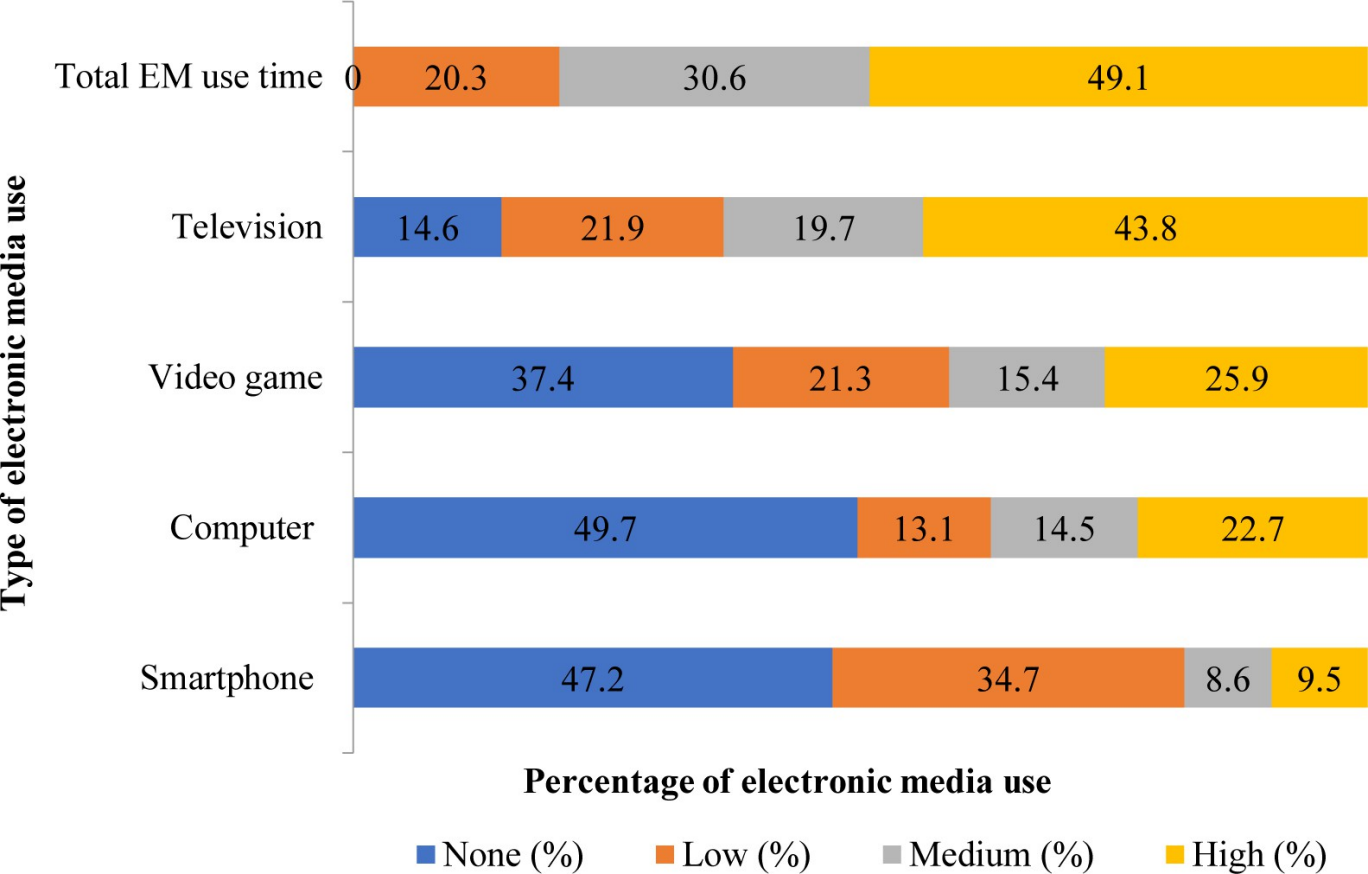
Adolescents spent time using electronic media (EM).

Adolescents spent time using electronic media (EM) presented in Fig 2. Among the total adolescent students, about 49.1% highly (above 3 hours per day) spent using electronic media whereas 30.6% and 20.3% were moderate (≥121 to 180 min/day) and low (<121 min/day) electronic media users. In this study, 19.7% and 43.8% of students moderately (≥69 to <115 min/day) and highly (≥115 min/day) watched television, 15.4%, and 25.9% were moderately (≥30 to 45 min/day) and highly (≥45 min/day) played video games, 14.5% and 22.7% moderately (≥30 to 45 min/day) and highly (≥45 min/day) used computer, 8.6% and 9.5% moderately (≥16 to <30 min/day) and highly (≥30 min/day) used smartphone. Moreover, 14.6%, 37.4%, 49.7%, and 47.2% reported not watching television, not playing video games, and not using the computer, and smartphone, respectively.

**Fig 3:**
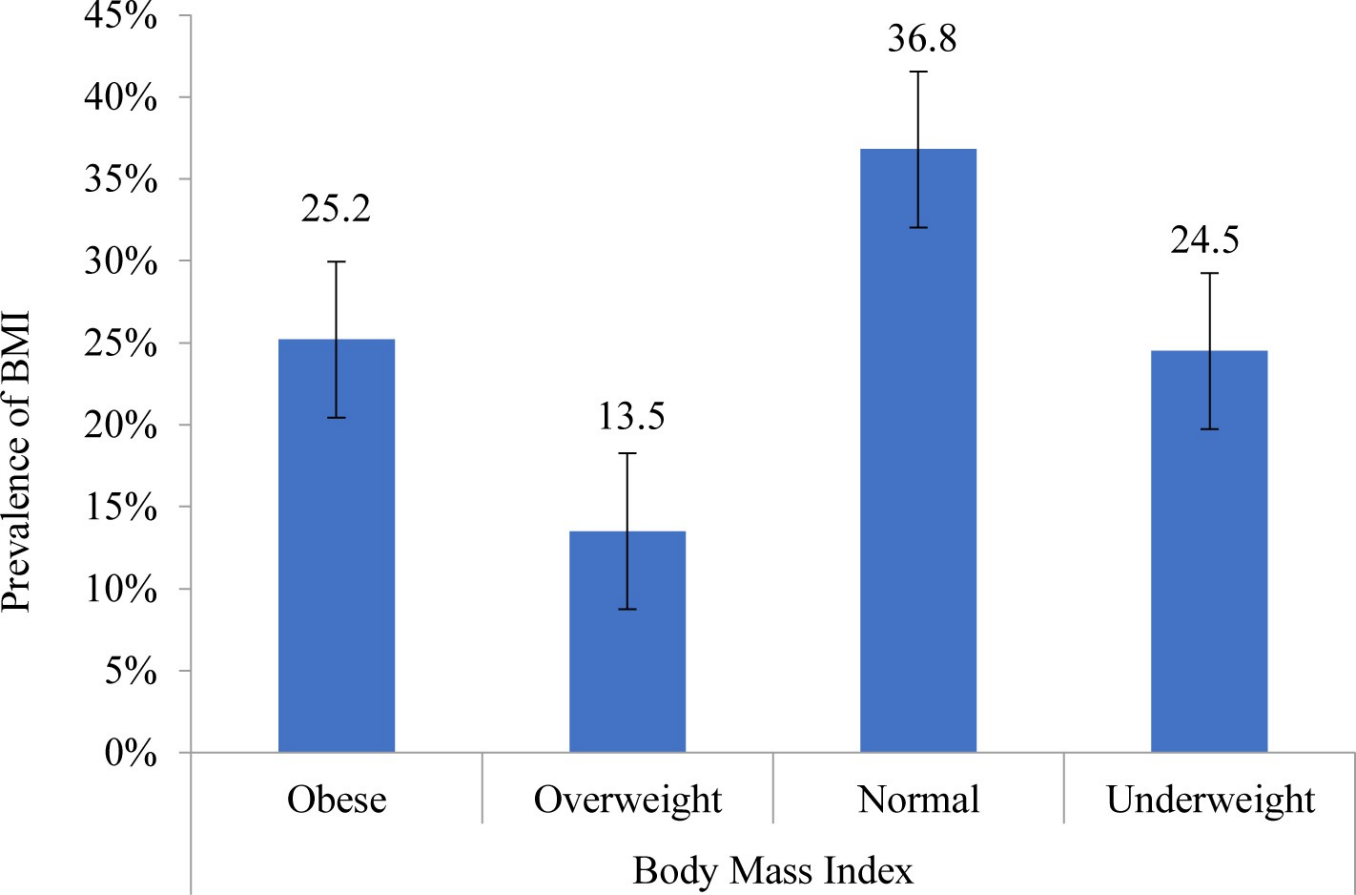
Anthropometric assessment of school adolescents.

These findings also explain that 25.2% were obese and 13.5% were overweight school adolescents while 36.8% were of normal weight (Fig 3). The prevalence of overweight and obesity increased with unhealthy eating habits during electronic media use and physical inactivity (*P*<0·05) (Table 2). This study further presented that the prevalence of overweight was considerably higher among adolescents who were moderately (15.6% vs 11.6%) and highly (17.2% vs 11.6%) use computer compared with those who were not (*P*<0·001). Again, a significantly higher proportion of the participants with moderate and high smartphone users had experienced being overweight and obese (*P*<0.05). The overweight and obesity prevalence were also significantly higher among moderate and high total media users than low media users (*P*<0·001).

**Table 2:** Bivariate analysis of exposure variable with BMI in Bangladeshi adolescents.

Exposure Variables		Body Mass Index				
	Total	Overweight (n = 47, 13.5%)	Obese (n = 88, 25.2%)	Normal (n = 129, 36.8%)	Under-weight (n = 86, 24.5%)	
	n (%)	%	%	%	%	*p-value*
**Gender**						
Boys	213 (60.8)	14.5	25.4	38.5	21.6	0.113
Girl	137 (39.2)	11.7	24.8	34.3	29.2	
**Class**						
Eight	150 (42.9)	14.0	25.3	34.7	26.0	0.891
Nine	200 (57.1)	13.0	25.0	38.5	23.5	
**Unhealthy eating habits during electronic media use**						
No	169 (48.3)	13.1	17.1	41.1	28.7	0.032
Yes	181 (51.7)	14.2	34.5	33.0	18.3	
**Physical exercise (≥30 min/day)**						
No	218 (62.4)	14.3	32.2	33.2	19.8	0.011
Yes	132 (37.6)	13.8	21.2	39.1	26.4	
**Television**						
None (0 min/day)	51 (14.6)	13.7	21.6	37.3	27.4	0.013
Low (<69 min/day)	76 (21.9)	15.7	35.3	26.9	22.1	
Moderate (≥69 to <115 min/day)	68 (19.7)	13.2	22.1	36.8	27.9	
High (≥115 min/day)	155 (43.8)	11.6	23.2	46.7	18.5	
**Video Games**						
None (0 min/day)	131 (37.4)	11.5	30.4	34.3	23.8	0.088
Low (>0 to <30 min/day)	74 (21.3)	14.8	27.1	31.1	27.0	
Moderate (≥30 to 45 min/day)	54 (15.4)	14.8	18.5	35.3	31.4	
High (≥45 min/day)	91 (25.9)	14.5	19.8	44.2	21.5	
**Computer**						
None (0 min/day)	173 (49.7)	11.6	27.3	33.7	27.4	<0.001
Low (>0 to <30 min/day)	45 (13.1)	11.1	15.5	46.6	26.8	
Moderate (≥30 to 45 min/day)	51 (14.5)	15.6	21.6	41.3	21.5	
High (≥45 min/day)	81 (22.7)	17.2	28.3	35.8	18.7	
**Smartphone use**						
None (0 min/day)	165 (47.2)	12.2	26.5	37.9	23.4	0.042
Low (>0 to <16 min/day)	121 (34.7)	11.6	18.8	40.7	28.9	
Moderate (≥16 to <30 min/day)	30 (8.6)	16.6	35.6	28.9	18.9	
High (≥30 min/day)	34 (9.5)	21.5	27.8	29.6	21.1	
**Total electronic media use**						
Low (<121 min/day)	71 (20.3)	9.8	17.8	51.7	20.7	<0.001
Moderate (≥121 to 180 min/day)	107 (30.6)	13.8	26.3	31.7	28.2	
High (≥181 min/day)	172 (49.1)	15.3	27.8	37.3	19.6	

The result estimates a significant association between screen-based electronic device use with overweight, and obesity (Table 3). This study presents the results in the form of relative risk ratios which report a change in the odds of overweight and obesity associated with a change in the level of explanatory variables. The present study revealed that EM use was a significant predictor of overweight among school adolescents. The regression model explains that the odds of being overweight were 2.53 (95% CI: 1.23–3.86) times higher among adolescents who had unhealthy eating habits during electronic media use than their counterparts having no unhealthy eating habits. Conversely, adolescents who performed physical exercise were 0.62 times less likely to be overweight compared with counterparts who had no physical activity. Adolescents who watched television at moderate and high levels were 2.12 (95% CI: 1.23–3.21) times and 4.58 (95% CI: 1.46–7.95) times more likely to be overweight relative to peers who did not watch television. Among adolescents, the odds of being overweight were 2.29 (95% CI: 1.25–3.64) times and 4.45 (95% CI: 2.75–6.12) times higher among moderately and highly played video games compared with those who did not play video games. Again, adolescents who reported a moderate and high level of computer use were more likely to be overweight (RRR: 2.21, 95% CI: 1.31–3.16; *P* = 0.02) and (RRR: 3.18, 95% CI: 1.87–4.70; *P* <0.001) than those who did not use the computer. The results also showed that adolescents with a high level of smartphone use were 2.15 times more likely to be overweight than those who did not use the smartphone. Additionally, in terms of total screen-based electronic devices usage, the odds of being overweight among moderate and high-level electronic media use were 4.92 (95% CI: 2.45–7.10) and 7.36 (95% CI: 3.64–11.54) times higher compared with those school adolescents who spent the least amount of time, respectively.

**Table 3:** Multinomial logistic regression for the association between electronic media use with BMI.

Exposure Variables	Body Mass Index	
	Overweight versus Normal	Obese versus Normal
	RRR (95% CI), *P*-value	RRR (95% CI), *P*-value
**Gender**		
Girl	Reference	Reference
Boys	0.92 (0.89–1.03), 0.54	0.83 (0.82–0.96), 0.42
**Class**		
Eight	Reference	Reference
Nine	1.44 (1.03–1.86), 0.07	1.31 (0.94–1.70), 0.53
**Unhealthy eating habits during electronic media use**		
No	Reference	Reference
Yes	2.53 (1.23–3.86), 0.001	2.40 (1.12–3.69), 0.001
**Physical exercise (≥30 min/day)**		
No	Reference	Reference
Yes	0.62 (0.54–0.71), 0.01	0.48 (0.43–0.56), 0.001
**Television**		
None (0 min/day)	Reference	Reference
Low (<69 min/day)	1.29 (0.56–2.18), 0.15	0.70 (0.33–1.08), 0.21
Moderate (≥69 to <115 min/day)	2.12 (1.23–3.21), 0.02	1.11 (0.53–2.06), 0.04
High (≥115 min/day)	4.58 (1.46–7.95), 0.04	2.89 (1.31–5.21), 0.03
**Video Games**		
None (0 min/day)	Reference	Reference
Low (>0 to <30 min/day)	0.85 (0.34–1.39), 0.64	0.90 (0.52–1.42)
Moderate (≥30 to 45 min/day)	2.29 (1.25–3.64), 0.03	1.69 (0.78–2.69), 0.04
High (≥45 min/day)	4.45 (2.75–6.12), 0.001	3.88 (1.74–5.13), 0.01
**Computer**		
None (0 min/day)	Reference	Reference
Low (>0 to <30 min/day)	0.76 (0.25–1.34), 0.66	0.86 (0.46–1.03), 0.72
Moderate (≥30 to 45 min/day)	2.21 (1.31–3.16), 0.02	1.91 (0.76–3.28), 0.03
High (≥45 min/day)	3.18 (1.87–4.70), <0.001	3.08 (1.32–4.86), <0.001
**Smartphone use**		
None (0 min/day)	Reference	Reference
Low (>0 to <16 min/day)	0.98 (0.57–1.40), 0.58	0.64 (0.25–1.14), 0.66
Moderate (≥16 to <30 min/day)	0.70 (0.37–1.14), 0.43	0.60 (0.31–1.09), 0.44
High (≥30 min/day)	2.15 (1.23–3.51), 0.001	1.19 (0.93–1.48), 0.002
**Total electronic media use**		
Low (<121 min/day)	Reference	Reference
Moderate (≥121 to 180 min/day)	4.92 (2.45–7.10), 0.03	6.52 (3.57–8.89), 0.04
High (≥181 min/day)	7.36 (3.64–11.54), <0.001	8.72 (4.64–12.54), <0.001

Note: The model was adjusted with gender, grade of school, unhealthy eating habits, and physical exercise variables.

The present study also explored the relationship between screen-based electronic device uses with obesity (Table 3). However, adolescents having unhealthy eating habits during electronic media use were 2.40 (95% CI: 1.12–3.69) times higher odds of obesity. Adolescents who performed physical exercise were 0.48 times less likely to be obese. The study findings also suggest that the odds of obesity among moderate and high-level television user adolescents were 1.11 (95% CI: 0.53–2.06, *P* = 0.04) times and 2.89 (95% CI: 1.31–5.21, *P* = 0.03) times more likely compared with those who did not use television. Regarding the association between video game use with obesity, adolescent students who reported a moderate and high level of video game use were 1.69 (95% CI: 0.78–2.69, *P* = 0.04) times and 3.88 (95% CI: 1.74–5.13, *P* = 0.01) times more likely to obese compared with peers those who did not play video games. Moreover, adolescents who used a computer at moderate and high levels were 1.91 times and 3.08 times more likely to be obese relative to peers who did not use a computer. The results also showed that adolescents with a high level of smartphone use were 1.19 times more likely to be obese than those who did not use the smartphone. In addition, the rate of obesity among school adolescents with a moderate and high level of total SED use was 6.52 times (RRR: 6.52, 95% CI: 3.57–8.89, *P* = 0.04) and 8.72 times (RRR: 8.72, 95% CI: 4.64–12.54, P<0.001) higher than those who spent the least amount of time.

## 4. Discussion

In this study, we examined the hypothesis that electronic media uses are implicated in the rising prevalence of overweight and obesity in Bangladeshi secondary school adolescents. Using multinomial logistic regression, this study found a significant multiplicative interaction between overweight and obesity with electronic media used among secondary school adolescents. In addition, the present study found that daily television viewing, playing video games, computer, and mobile phone use, and total electronic devices use time was associated with overweight and obesity.

In the current study, unhealthy eating habits during electronic media use were significantly associated with overweight and obesity. The previous findings from other countries are in a line with our findings [12, 35, 36]. This could be due to prolonged sedentary behavior and high energy intake may promote visceral fat in adolescents [37]. Unhealthy eating habits including diets that are higher in fat, and drink in more sodas, and fast food during electronic device use may lead to overconsumption among school adolescents [35, 38]. In agreement with previous experimental studies [39, 40], the present study found that physical exercise was inversely associated with overweight and obesity in secondary school adolescents. Recently, different studies explained that excess screen time in adolescents, increased sedentary time, and unhealthy dietary behaviors are also associated with physical inactivity [41, 42].

This study also found that excessive TV viewing was associated with overweight and obesity among school adolescents. The strength of these associations is consistent with those found in previous studies [18, 43, 44]. Other studies conducted in different countries have confirmed that watching television is positively associated with overweight and obesity in school adolescents [45–47]. From the evidence, though it has been proved that long hours of watching TV are associated with adolescent obesity [18, 45], all screen-based electronic media should be considered attention.

Furthermore, this study reported that the use of video games was significantly associated with school adolescents being overweight and obesity. This finding is in line with an earlier study that found adolescents with a longer time playing electronic games daily were increased risk of obesity [13]. Prior studies also revealed that increased hours of playing video games increased the risk of adolescents being overweight and obesity [13, 47]. The main reason for increasing weight among young adolescents is that they play video games as a substitute for regular physical exercise which expenditure less energy than required [13, 48].

Another important finding of this study is that heavy use of computer was significantly associated with overweight and obesity. Several studies have also linked a stronger association between computer use and obesity in adolescents [14, 47]. Besides, recent studies claim that the likelihood of obesity is higher among young adolescents with heavy computer use [17, 22, 44, 49]. This study revealed that obesity increased in adolescents who spent high time on a smartphone compared with non-user adolescents in all the survey periods. The findings of the study concerning the relationship between smartphone use and obesity are consistent with prior studies [13, 14, 22]. Finally, the present study revealed that overall electronic media usage was a significant predictor of overweight and obesity among school adolescents. Additionally, the current study results also indicate that spending above 2 hours daily on any electronic media device was significantly associated with increased odds of being overweight and obese among school adolescents. This finding is also consistent with other previous research suggesting that the use of electronic media is associated with adolescent overweight and obesity [4, 15, 50, 51].

There are several possible justifications for our study findings that explain the effects of electronic media use on obesity in adolescents [35, 36]. Firstly, the displacement of physical activity with sedentary leisure-time use increased the adolescent’s BMI [35, 36, 46]. Secondly, it is also important to consider that electronic media use affects the dietary choice and more energy consumption which may contribute to adolescent obesity [35, 36]. Some epidemiological studies explain that heavy electronic media can lead to consuming more energy (i.e., energy-dense snacks, energy-dense drinks, and fast food while viewing) [12, 19]. Additionally, another explanation for the association between electronic media use and obesity is food advertising which led to consuming more energy among young adolescents [52, 53]. Finally, inadequate sleep is another possible explanation associated with electronic media use, excess energy consumption, and obesity [35].

To the best of our knowledge, this is the first population-based study that investigates the pattern of the time usage of common electronic devices among Bangladeshi secondary school adolescents and explores the association between overweight, and obesity with electronic media use. This finding suggests that using electronic media (EM) at a specific level would be a risk factor for increasing overweight and obesity in these young adolescents. In addition, further longitudinal studies are needed to investigate the detailed pattern of EM usage and its associations with BMI.

Moreover, the current findings recommend effective interventions which can be designed to reduce overweight in-school adolescents and improve their health status. The types of behavioral interventions including classroom-based physical education lessons, changing school diet patterns in the school cafeteria, and automated screen monitoring devices to control screen time at school and home may provide useful evidence to adolescents for reducing their weight [54–56]. Additionally, active video games and healthy diet-related videos have the potential to improve body composition in overweight and obese adolescents [57, 58]. Nowadays, involving more common physical activity, healthy dietary choices, and minimizing the recreational use of EM could be implemented for managing a healthy weight. This study also may provide useful evidence to adolescent health policymakers and parents to initiate adolescent health-based interventions to reduce obesity among young adolescents and thus improve their health status.

This study has some potential limitations that should be considered. First, the study results might be vulnerable to self-reported bias, as data on media use and other covariates were self-reported. Second, this study also covered a limited area and the sample was convenient which may limit generalization. Furthermore, this study is a cross-sectional design, which does not clarify the causal directions of the relationships. Longitudinal studies are essential to confirm our results. This study might be considered a robust method to find descriptive statistics and identify new promising problems as a result of their low budget and high feasibility.

## 5. Conclusion

This study reveals that adolescents who used one or more types of screen-based electronic devices for more than two hours per day were more likely to be overweight and obese, but this association varies depending on the type of electronic device used. The study found that television watching was found to be associated with the risk of overweight and obesity was higher among school-going adolescents. This study also revealed that video game playing, computer, and smartphone use were associated with overweight and obesity among adolescents. However, this study strongly encourages the future to conduct longitudinal studies to confirm our findings and establish the trend of correlation with weight status. Implementation of school-based intervention programs may effectively reduce the use of EM among youth adolescents. Besides, parents should play an important role to reduce the time their children spend using electronic devices and encourage them to engage in physical activities.

## Supporting information

S1 File The questionnaire template is available in both English and the native language. (DOCX)

## Abbreviations

EM: Electronic media
WHO: World Health Organization
BMI: Body Mass Index
MARCA: Multimedia Activity Recall for Children and Adolescents
RRR: Relative Risk Ratio
CI: Confidence Interval
