# Supplementary material for: The degree of association between overweight and obesity with the use of electronic media among Bangladeshi adolescents
Source: PLoS One. 2023 Jan 20;18(1):e0280544. doi: 10.1371/journal.pone.0280544 (PMC9858059; doi:10.1371/journal.pone.0280544)
Supplement: S1 File — (DOCX) [file pone.0280544.s001.docx]

**Questionnaire English Version**

**The degree of association of overweight and obesity with the use of electronic media among Bangladeshi adolescents**

1. **Background characteristics of study participants**

| **SN.** | **Variables** | **Categories** | | | |
| --- | --- | --- | --- | --- | --- |
| **1** | Gender | (a) Boy | (b) Girl |  |  |
| **2** | Grade of school | (a) Eight | (b) Nine |  |  |
| **3** | Mother educational level | (a) Primary | (b) Secondary | (c) Higher Secondary | (d) Graduation degree |
| **4** | Father educational level | (a) Primary | (b) Secondary | (c) Higher Secondary | (d) Graduation degree |
| **5** | Mother occupation | (a) Housewife | (b) Govt. Employee | (c) Private Employee | (d) Business |
| **6** | Father occupation | (a) Govt. Employee | (b) Private Employee | (c) Business | (d) Day labor |
| **7** | Monthly family income | (a) <10,000 BDT | (b) 11,000-20,000 BDT | (c) 21,000-30,000 BDT | (d) >31,000 BDT |
| **8** | Unhealthy eating habits during electronic device use | (a) Yes | (b) No |  |  |
| **9** | Physical exercise (≥30 min/day) | (a) Yes | (b) No |  |  |

1. **Anthropometric Assessment**

| **1.** | **(a) Height (inches)** |  | **(b) Weight (kg)** |  | **(c) BMI** |  |
| --- | --- | --- | --- | --- | --- | --- |

1. **Screen Based Electronic Media Use**

| **SN.** | **Variables** | **Categories** | | | |
| --- | --- | --- | --- | --- | --- |
| **1** | **Television** | (a) None (0 min/day) | (b) Low (<69 min/day) | (c) Moderate (≥69 to <115 min/day) | (d) High (≥115 min/day) |
| **2** | **Video Games** | (a) None (0 min/day) | (b) Low (>0 to <30 min/day) | (c) Moderate (≥30 to 45 min/day) | (d) High (≥45 min/day) |
| **3** | **Computer** | (a) None (0 min/day) | (b) Low (>0 to <30 min/day) | (c) Moderate (≥30 to 45 min/day) | (d) High (≥45 min/day) |
| **4** | **Smartphone use** | (a) None (0 min/day) | (b) Low (>0 to <16 min/day) | (c) Moderate (≥16 to <30 min/day) | (d) High (≥30 min/day) |
| **5** | **Total electronic media use** | (a) None (0 min/day) | (b) Low (<121 min/day) | (c) Moderate (≥121 to 180 min/day) | (d) High (≥181 min/day) |

**Questionnaire Bengali Version (প্রশ্নাবলী বাংলা সংস্করণ)**

**বাংলাদেশী কিশোর-কিশোরীদের মধ্যে ইলেকট্রনিক মিডিয়া ব্যবহারের সাথে অতিরিক্ত ওজন এবং স্থূলতার সম্পর্ক (The degree of association of overweight and obesity with the use of electronic media among Bangladeshi adolescents)**

1. **অধ্যয়ন অংশগ্রহণকারীদের পটভূমি বৈশিষ্ট্য**

| **ক্রমিক সংখ্যা** | **ভেরিয়েবল** | **ক্যাটাগরি** | | | |
| --- | --- | --- | --- | --- | --- |
| **১** | **লিঙ্গ** | (ক) ছেলে | (খ) মেয়ে |  |  |
| **২** | **স্কুলের গ্রেড** | (ক) অষ্টম | (খ) নবম |  |  |
| **৩** | **বয়স** | (ক) ১৪ | (খ) ১৫ | (গ) ১৬ |  |
| **৪** | **মায়ের শিক্ষাগত যোগ্যতা** | (ক) প্রাথমিক | (খ) মাধ্যমিক | (গ) উচ্চ মাধ্যমিক | (ঘ) স্নাতক ডিগ্রি |
| **৫** | **পিতার শিক্ষাগত যোগ্যতা** | (ক) প্রাথমিক | (খ) মাধ্যমিক | (গ) উচ্চ মাধ্যমিক | (ঘ) স্নাতক ডিগ্রি |
| **৬** | **মায়ের পেশা** | (ক) গৃহিণী | (খ) সরকারি কর্মচারী | (গ) বেসরকারি কর্মচারী | (ঘ) ব্যবসায়ী |
| **৭** | **পিতার পেশা** | (ক) সরকারি কর্মচারী | (খ) বেসরকারি কর্মচারী | (গ) ব্যবসায়ী | (ঘ) দিনমজুর |
| **৮** | **মাসিক পারিবারিক আয়** | (ক) <১০,০০০ টাকা | (খ) ১১,০০০ - ২০,০০০ টাকা | (গ) ২১,০০০ - ৩০,০০০ টাকা | (ঘ) >৩১,০০০ টাকা |
| **৯** | **ইলেকট্রনিক ডিভাইস ব্যবহারের সময় অস্বাস্থ্যকর খাদ্যাভ্যাস** | (ক) হ্যাঁ | (খ) না |  |  |
| **১০** | **শারীরিক ব্যায়াম (≥৩০ মিনিট/দিন)** | (ক) হ্যাঁ | (খ) না |  |  |

1. **নৃতাত্ত্বিক পরিমাপ**

| **১** | **(ক) উচ্চতা (ইঞ্চি)** |  | **(খ) ওজন (কেজি)** |  | **(গ)** **বিএমআই** |  |
| --- | --- | --- | --- | --- | --- | --- |

**C. স্ক্রীন ভিত্তিক ইলেকট্রনিক মিডিয়া ব্যবহার**

| **ক্রমিক সংখ্যা** | **ভেরিয়েবল** | **ক্যাটাগরি** | | | |
| --- | --- | --- | --- | --- | --- |
| **১** | **টেলিভিশন** | (ক) কোনটিই নয় (০ মিনিট/দিন) | (খ) কম (<৬৯ মিনিট/দিন) | (গ) মাঝারি (≥৬৯ থেকে < ১১৫ মিনিট/দিন) | (ঘ) উচ্চ (≥১১৫ মিনিট/দিন) |
| **২** | **ভিডিও গেমস** | (ক) কোনটিই নয় (০ মিনিট/দিন) | (খ) কম (>০ থেকে <৩০ মিনিট/দিন) | (গ) মাঝারি (≥৩০ থেকে ৪৫ মিনিট/দিন) | (ঘ) উচ্চ (≥৪৫ মিনিট/দিন) |
| **৩** | **কম্পিউটার** | (ক) কোনটিই নয় (০ মিনিট/দিন) | (খ) কম (>০ থেকে <৩০ মিনিট/দিন) | (গ) মাঝারি (≥৩০ থেকে ৪৫ মিনিট/দিন) | (ঘ) উচ্চ (≥৪৫ মিনিট/দিন) |
| **৪** | **স্মার্টফোন ব্যবহার** | (ক) কোনটিই নয় (০ মিনিট/দিন) | (খ) কম (>০ থেকে <১৬ মিনিট/দিন) | (গ) মাঝারি (≥১৬ থেকে <৩০ মিনিট/দিন) | (ঘ) উচ্চ (≥৩০ মিনিট/দিন) |
| **৫** | **মোট ইলেকট্রনিক মিডিয়া ব্যবহার** | (ক) কোনটিই নয় (০ মিনিট/দিন) | (খ) কম (<১২১  মিনিট/দিন) | (গ) মাঝারি (≥১২১ থেকে ১৮০ মিনিট/দিন) | (ঘ) উচ্চ (≥১৮১ মিনিট/দিন) |
